# Social relationships, living arrangements and loneliness

**DOI:** 10.1007/s00391-021-01960-1

**Published:** 2021-08-20

**Authors:** W. Schmitz, S. Mauritz, M. Wagner

**Affiliations:** 1 Institute of Gerontology, Department of Ageing and Work, University of Vechta, Driverstr. 23, 49377 Vechta, Germany; 2 Graduate School GROW, Gerontological Research On Well-Being, University of Cologne, Cologne, Germany; 3 Institute of Sociology and Social Psychology, University of Cologne, Cologne, Germany

**Keywords:** Social network, LAT partnership, Coresidential partnership, Very old age, Germany, Soziales Netzwerk, LAT Partnerschaft, Coresidentielle Partnerschaft, Hochaltrigkeit, Deutschland

## Abstract

**Background:**

Oldest-old people are expected to be particularly likely to experience loneliness due to the loss of their intimate partner or of same-aged social network members. It is assumed that individuals in different living arrangements maintain different kinds of social networks because they adjust their networks to their specific needs. However, not much is known about the variation in the social networks of the oldest-old depending on their living arrangements and how this variation is related to loneliness. This is the first study that seeks to fill this research gap by examining how the composition and the size of a social network varies among the oldest-old depending on their living arrangements with a partner (coresidential partnership, living apart together (LAT) partnership, no partnership), and how this variation contributes to explain loneliness among the oldest-old.

**Methods:**

We used cross-sectional data from the representative survey NRW80+ (Quality of Life and Well-Being of the Very Old in North-Rhine Westphalia). The sample of analysis used in this study consists of 1860 respondents from the German state of North-Rhine Westphalia aged 80 years and older. Associations between social network characteristics and living arrangements were tested using χ^2^-tests and one-way ANOVA. Ordered logit models were used to explain loneliness.

**Results:**

Respondents in a coresidential partnership maintained larger social networks than those in an LAT partnership and those with no intimate partner. Furthermore, the respondents with no partner maintained more diverse social networks. Compared to those in the other living arrangements, the respondents in an LAT partnership maintained the smallest and least diverse social networks. Being in a coresidential partnership and the social network size were found to be negatively associated with loneliness.

**Conclusion:**

First, the results indicate that respondents who do not have a partner adjusted their social networks to meet their needs in the absence of this relationship. Second, we conclude that being in a coresidential partnership and having a large social network protects the oldest-old against loneliness.

**Supplementary Information:**

The online version of this article (10.1007/s00391-021-01960-1) contains supplementary material, which is available to authorized users.

## Abstract (de)

**Hintergrund:**

Es ist zu erwarten, dass insbesondere Hochaltrige von Einsamkeit betroffen sind, da sie mit höherer Wahrscheinlichkeit den eigenen Partner oder gleichaltrige soziale Netzwerkmitglieder verlieren. Es wird angenommen, dass sich Individuen in verschiedenen Lebensformen hinsichtlich ihres sozialen Netzwerks unterscheiden, da dieses an ihre unterschiedlichen Bedürfnisse angepasst ist. Dennoch ist wenig darüber bekannt, wie sich das soziale Netzwerk zwischen coresidentiellen Partnerschaften, „living-apart-together“ (LAT) Partnerschaften, sowie Partnerlosen unterscheidet und wie dies mit Einsamkeit zusammenhängt. Dieses ist die erste Studie, in der untersucht wird, wie das soziale Netzwerk nach partnerschaftlichen Lebensformen variiert und wie dieses Einsamkeit im hohen Alter erklärt.

**Methoden:**

Wir nutzen Querschnittsdaten aus der Repräsentativitätsstudie NRW80+ (Lebensqualität und Wohlbefinden hochaltriger Menschen in Nordrhein-Westfalen). Das Analysesample dieser Studie besteht aus 1860 Befragten aus Nordrhein-Westfalen im Alter ≥80 Jahre. Zusammenhänge zwischen der Größe und Komposition des sozialen Netzwerks und den Lebensformen werden mittels Chi-Quadrat-Tests und einfaktoriellen Varianzanalysen (ANOVA) getestet. Logistische Regressionsmodelle werden genutzt, um Einsamkeit im hohen Alter zu erklären.

**Ergebnisse:**

Personen in coresidentiellen Partnerschaften weisen ein größeres soziales Netzwerk auf als Personen in LAT-Partnerschaften und Personen ohne Partner. Personen ohne Partner sind hingegen in ein vielfältigeres soziales Netzwerk integriert. Hochaltrige in LAT-Partnerschaften zeigen das kleinste und wenig diverseste soziale Netzwerk im Vergleich zu den beiden anderen Lebensformen. Coresidentielle Partnerschaften und die Größe des sozialen Netzwerks hängen negativ mit Einsamkeit zusammen.

**Diskussion:**

Zum einen legen unsere Ergebnisse nahe, dass Hochaltrige ohne Partnerschaft das eigene soziale Netzwerk ihren Bedürfnissen entsprechend anpassen, um das Fehlen eines Partners auszugleichen. Zum anderen schließen wir aus den Befunden, dass das Leben in einer coresidentiellen Partnerschaft und ein größeres soziales Netzwerk vor Einsamkeit im hohen Alter schützen.

## Introduction

Social relationships are crucial for the well-being of the very old. Two sociological concepts are essential to describe the structures of social relationships: social networks and living arrangements. While the social network can be described by its functional (e.g. social support) and its structural characteristics (e.g. size, composition) [8], this article focuses on the latter. Living arrangements are understood as patterns of social relationships with people with whom the individuals live and share their everyday lives [18]. Most notably, the relationship with an intimate partner is strongly associated with well-being in old age, as having a partner can prevent loneliness [7, 24, 28]. Loneliness is defined as the feeling that there is a discrepancy between the actual and the desired quality and quantity of an individual’s social relationships. The feeling of missing an attachment figure (e.g., intimate partner) is defined as emotional loneliness, whereas the feeling of missing a broader social network (e.g., friends) is understood as social loneliness [17]. Overall, loneliness is known to jeopardize a person’s health and well-being [12]. This article examines the association between an individual’s egocentric social network, which describes the connections between the individual and a set of other people [8] and the person’s living arrangement with an intimate partner. Moreover, we investigate how the structure of the social relationships of very old people influences their feelings of loneliness. Thus, based on the framework “Challenges and Potentials Model of Quality of Life in Very Old Age (CHAPO)” [29], we focus on the social conditions that underlie successful life conduct.

Although feelings of loneliness increase slightly after the age of 70 years, especially for women, the overall risk of experiencing loneliness is low among the oldest old [14]. Based on socioemotional selectivity theory, a possible explanation for the low risk of loneliness in very old age is that only a few close relationships, such as the relationship with a partner, might be satisfying enough for the emotional needs of the oldest old [5]. Living with a partner is associated with experiencing fewer feelings of loneliness [28], whereas living alone and being widowed are substantial risk factors for experiencing loneliness [2, 9]; however, the proportion of people who are widowed or are living without a partner are higher among those aged ≥80 years than they are among younger age groups [19, 23, 26]. Among people aged 80+ years, 70% of men and only 20% of women are living in a coresidential partnership [19]. In addition, very old people who have a partner are especially likely to live in a living apart together (LAT) partnership. This living arrangement may be seen as an alternative to forming a more institutionalized partnership after the loss of a spouse [16], or it may occur because one of the former coresidential partners has moved to an institutional care setting [22].

Moreover, it is well-known that social inequality, the type of living arrangement and loneliness are related to each other. For example, among the oldest old, higher educated men are more likely to be in a coresidential partnership than lower educated men [19]. Higher educated persons are also less likely to feel lonely [25].

A possible strategy for dealing with the challenges of being in an LAT partnership or of having no intimate partner (e.g., the lack of social support) is to extend the social network. The hierarchical compensatory model assumes that individuals compensate for the loss or absence of a potential social support source by becoming more involved with other social network members (e.g., children) [4]. Thus, individuals who live apart from their partner or who lack a partner may be expected to have a different set of social relationships than people who are in a coresidential partnership; however, the empirical results on this issue are fragmentary, and are often focused on marital status. It has been shown that among oldest old people in Germany, those who have no partner maintain a more diverse social network by including more non-kin relationships in their network, whereas those who have a partner report having a larger network size [15, 30]. Moreover, after people are widowed their contact to all types of network members, particularly to children and siblings, tends to increase [10].

For older people without a partner, their children, grandchildren, and siblings are especially valuable sources of social support [27]. However, having a non-kin network of friends and acquaintances as well as a bigger social network size is also important for older people as these relationships enable them to engage in social activities and to exchange information [1]. In line with the hierarchical compensatory model, having children, siblings, friends, and neighbors provides greater protection from loneliness for unmarried people than for married people [24]. Nonetheless, older people who lack a partner and who live with their children or with other family members are as likely to report feeling lonely as older people who live in a single person household [9]. Overall, the number of friends and acquaintances people have is more strongly associated with loneliness than the number of family members they have [25].

However, most research on this topic focuses either on social networks or on living arrangements but does not view them as mutually dependent. Thus, up to now, it is largely unknown how the social networks of the oldest old vary depending on their living arrangements. Most of these studies focus on marital and coresidential partnerships [19, 24, 25], or concentrate on living alone as a risk factor of loneliness, without considering other kinds of living arrangements with the partner [2, 28]. Moreover, little is known about the association of loneliness with various relationship types among the oldest old, because most studies only examine single relationship characteristics like the number of friends or children [20].

Our study aims to fill this research gap by examining how the living arrangements of the oldest old are related to their social embeddedness and to what extent both their living arrangements and social embeddedness are associated with loneliness. By contributing representative insights into these issues and considering different living arrangements of the oldest old, this study seeks to tackle the stated shortcomings of previous research.

## Methods and measurements

In this study, we used cross-sectional data from the representative survey “Quality of Life and Well-Being of the Very Old in North Rhine-Westphalia” [29] (NRW80+, *n* = 1863). As we dropped 3 cases because of ambiguous information, the final sample consists of 1860 cases, including 176 proxy interviews and 211 interviews with nursing home residents. The shares of missing values were highest for the variables on depression (7.3%) and education (7.2%). We used multiple imputation to substitute missing values for 323 observations (17.4%). All variables with missing values were imputed. Since gender has no missing values, the variable was not imputed. The imputation model was predicted by gender and type of residence (institutional care/private household). Both the imputed and the original dataset led to the same results.

In the following, we present a descriptive overview of the respondents’ living arrangements by the size and composition of their social networks and their levels of education. These associations are tested using one-way ANOVA and corrected weighted Pearson χ^2^ statistics. In a second step, we use ordered logit models to examine how the respondents’ social network sizes, relationship types, and living arrangements are related to their levels of loneliness. We use depression, education, gender, and age as controls. All statistical models and descriptive statistics are adjusted for the two-stage survey design of our data.

The social network size was measured by asking each respondent for the names of up to four of the most important people in his/her life and varied between zero and four. Furthermore, the respondents could specify the type of social relationship (e.g., partner, children) for each of these individuals. For each relationship type, we generated a dichotomous variable indicating if the relationship type was reported or not. We distinguish between five categories: children and grandchildren, siblings, other family members, friends and acquaintances. The respondents’ living arrangements were measured by their partnership status, household composition, and type of residence. If a respondent reported the presence of a partner when asked about the household composition, the respondent was considered to be in a coresidential partnership. If a respondent did not list a partner when asked about the household, or stated that they lived in institutional care, the respondent was considered to be in an LAT partnership [22]. Therefore, we can differentiate between three living arrangements: a coresidential partnership, an LAT partnership, and living without a partner. Loneliness was measured by the item: “how often did you feel lonely in the last week?”, with an ordinal scale ranging from one to four. Higher values mean that the respondent felt lonely more frequently. The levels of depression were measured using four dichotomous items (e.g., can enjoy life), which were summed up and ranged from one to four, with higher values indicating higher levels of depressive symptoms. Education was measured by differentiating between three levels: level 1 includes primary or lower secondary education; level 2 includes upper secondary or post-secondary non-tertiary education; and level 3 indicates those with a bachelor, master, or doctoral degree or equivalent. More details on the descriptive characteristics of the sample (Table A) are provided in Supplement 1.

## Results

Table 1 describes the living arrangements by network size, network composition, and educational levels. The last column depicts the F‑statistics, which tested (1) the association between living arrangements and social network relationship types and educational levels; and (2) the differences in the means of the social network size by living arrangements. The results show that the respondents who were in a coresidential partnership maintained larger social networks than the respondents who had no partner, and that the respondents who were in an LAT partnership had the smallest networks. No significant associations can be found between the respondents’ living arrangements and whether there were children, grandchildren, or friends in their social networks.

**Table 1:** Distribution of social network characteristics and educational level by living arrangements

	**Living arrangement**			
	Coresidential partnership	Living apart together partnership	No partnership	
	M (SE), %			F
*Social network*				
Size	3.4 (0.1)	2.7 (0.2)	3.0 (0.1)	26.3***
Children and grandchildren	79.0	66.4	75.4	2.7
Siblings	11.2	5.4	16.4	6.4**
Other family members	25.1	30.1	38.8	13.4***
Friends	13.0	11.2	16.4	1.6
Acquaintances	10.4	10.2	20.2	10.1***
*Education*	–	–	–	18.9***
Low	16.2	22.1	35.6	–
Intermediate	56.6	53.7	50.7	–
High	27.2	24.3	13.7	–
Total	35.5	5.2	59.3	–
*N*	660	97	1103	–

*M* Mean, *SE* Standard error

NRW80+; *n* = 1860; weighted data; * *p* < 0.05; ** *p* < 0.01; *** *p* < 0.001

However, the respondents who had no partner were more likely than those in other living arrangements to list siblings, other family members, or acquaintances as social network members. Significant educational differences can also be observed between the respondents in different living arrangements. The proportion of people who were highly educated was greater among those who were living in a coresidential partnership, whereas the respondents with low levels of education were more likely to have no partner.

The results of the ordered logit models for loneliness are shown in Table 2. Results for control variables (depression, age, gender, education) and the thresholds (Table B) are provided in Supplement 2. In model 1, we found a significant association between loneliness and being in a coresidential partnership. The respondents living in a coresidential partnership were less likely to report feeling lonely than those who did not have an intimate partner. The respondents who were in an LAT relationship, by contrast, did not seem to differ in their levels of loneliness from those who had no partner. Model 2 also included the size of the social network, which improved the model fit (F = 11.42, *p* = 0.001). We found that having a larger social network size was associated with a lower likelihood of feeling lonely. The previously described association between being in a coresidential partnership and loneliness remained significant when the social network size was included. Additionally, we found that more depressive symptoms and increasing age are related to a higher risk of loneliness.

**Table 2:** Results of ordered logistic regression for loneliness

	Model 1		Model 2	
Variable	β (SE)	95% CI	β (SE)	95% .I
*Living arrangement* (Ref. No partner)				
Coresidential partnership	−1.39*** (0.17)	−1.73 −1.05	−1.25*** (0.17)	−1.58 −0.91
LAT partnership	0.14 (0.30)	−0.45 0.74	0.20 (0.30)	−0.40 0.80
*Social network*				
Size	–	–	−0.24** (0.07)	−0.37 −0.10
Children and grandchildren	−0.24 (0.16)	−0.56 0.08	0.14 (0.20)	−0.26 0.53
Siblings	−0.26 (0.25)	−0.74 0.23	−0.08 (0.26)	−0.59 0.43
Other family members	−0.13 (0.15)	−0.42 0.16	0.08 (0.15)	−0.21 0.38
Friends	−0.19 (0.22)	−0.62 0.24	−0.02 (0.22)	−0.46 0.42
Acquaintances	−0.33 (0.20)	−0.73 0.07	−0.14 (0.21)	−0.56 0.27
F	19.32***	–	19.34***	–
*N*	1860	–	1860	–

*SE* Standard error, *CI* Confidence interval, *LAT* Living apart together, *β* Unstandardized coefficient

Note: NRW80+; weighted data; Both models control for depression, age, gender and education

** *p* < 0.01; *** *p* < 0.001

## Discussion

This study has provided an overview of the living arrangements and social networks among the oldest old population in the most-populated state of Germany, and their associations with loneliness. Using representative cross-sectional data of respondents aged ≥80 years we found evidence that individuals without a partner coped with the lack of a partnership by increasing their investments in alternate relationship types. In line with the assumptions of the hierarchical compensatory model, our results indicated that compared to people in other living arrangements, individuals without a partner maintained a shifted hierarchy of social relationships with a broader range of social relationship types, such as relationships with siblings, other family members, and acquaintances.

However, we also found that compared to their counterparts in other living arrangements, the respondents who were in a coresidential partnership had larger social networks. This finding contradicts the claims of the hierarchical compensatory model. We assume that compared to the individuals who were in an LAT partnership or who had no partner, those in a coresidential partnership were more likely to be introduced to new social relationships through their partner. Moreover, the respondents in a coresidential partnership might have been more integrated into a broader family with children.

Furthermore, compared to the respondents in other types of living arrangements, those who were in a coresidential partnership had higher levels of education, in line with previous research [16, 19]. We assume that people with less education were more likely to have experienced the death of a spouse or to have moved to an institutional care setting [21].

Moreover, we found that compared to the individuals in other living arrangements, the respondents who were in an LAT partnership had both a smaller network size and a less diverse network composition. A possible explanation for this finding is that some of these oldest old people were living in an institutional care setting where they were no longer in close proximity to their former community, which impedes personal contact with their network members.

One of our main findings is the association between living in a coresidential partnership, having a larger social network size and being less lonely. People living in an LAT partnership were as lonely as those who had no partner. In light of these findings, our first conclusion is that when seeking to prevent loneliness among the oldest old, it is crucial to take into account whether an individual shares his/her household with a partner. Second, we speculate that having a larger number of social network members provides the oldest old with more access to social support and opportunities to engage in social activities, which may result in less loneliness [7]. Finally, we note that the strong association we found between being in a coresidential partnership and loneliness can also be interpreted in reference to the socioemotional selectivity theory, which states that the oldest old find emotionally close relationships the most rewarding [5]. Our findings on the association between partnership status, the size and the composition of the social network, and loneliness are supported by previous research [6, 7, 24, 25]. Additionally, we found that age and depression are related to loneliness among the oldest old. We, therefore, conclude that feelings of loneliness are more likely with increasing age [14] and that experiencing depressive symptoms might strengthen feelings of loneliness [3].

However, our study was unable to identify the mechanisms (e.g., the preferences and needs of the individuals) that underlie the associations between living arrangements, social network characteristics, and loneliness. Moreover, we had no information on the quality of each social relationship type. Prior research has shown that loneliness differs by the perceived quality and quantity of social relationships [13, 24]. Thus, having a partner does not necessarily result in better well-being, because this association depends for example on the satisfaction with the reciprocity of the relationship [11]. Furthermore, as the data did not include information on types of social relationships for more than four people, we were unable to draw a full picture of the composition of the social networks among the oldest old people in our sample, especially for the respondents who named their partner as one of the four social network members. Finally, as our study was based on cross-sectional data, we were unable to draw causal conclusions. These limitations should be addressed in future research.

## Conclusion with practical recommendations

- Having a coresidential partner and being socially embedded in a larger number of social relationships can prevent loneliness.
- People who have no partner or who are in an LAT relationship are at additional risk of loneliness because they tend to have a smaller social network than people who have a coresidential partner.
- For the oldest old who lack a partner or who are in an LAT relationship, interventions designed to enhance their opportunities to establish new social contacts are needed.

## Supplementary Information

Supplement 1: Table A. Descriptive characteristics of the sample by age and gender

Supplement 2: Table B. Results of ordered logistic regression for loneliness

## Declarations

All procedures performed in studies involving human participants or on human tissue were in accordance with the ethical standards of the institutional and/or national research committee and with the 1975 Helsinki declaration and its later amendments or comparable ethical standards. Informed consent was obtained from all individual participants included in the study.
