# Supplementary material for: Social relationships, living arrangements and loneliness
Source: Z Gerontol Geriatr. 2021 Aug 20;54(Suppl 2):120–5. doi: 10.1007/s00391-021-01960-1 (PMC8551096; doi:10.1007/s00391-021-01960-1)
Supplement: Supplementary file 1 — Supplement 1: Table A. Descriptive characteristics of the sample by age and gender [file 391_2021_1960_MOESM1_ESM.docx]

**Supplement 1**

*Supplementary information on descriptive characteristics of the sample*

Table A depicts the weighted sample characteristics by age and gender. Living arrangements varied strongly by age and gender. Coresidential partnerships were more common among younger age groups and among men. Moreover, whereas men and women were almost equally likely to be in an LAT partnership in the age group of 80 to 84 years, the gender differences in the frequences of being in such a partnership rose with increasing age. Men in higher age groups lived in an LAT partnership more frequently, while women much more frequently had no partner. However, the proportion of respondents who had no intimate partner was higher in the older age groups for both men and women compared to younger age groups. Looking at the social network characteristics, it is apparent that the respondents in the younger age groups had larger social networks. In addition, the composition of the respondents’ social networks differed by gender and age. For example, women listed family members and friends more frequently as members of their networks than men, especially in higher age groups. Acquaintances were listed more frequently as network members among respondents aged 85 and older. While most of the oldest-old respondents had an intermediate level of education, women had, on average, lower educational levels than men. In total, the prevalence of loneliness and depressive symptoms among the respondents was low. However, both loneliness and depression were more prevalent among women in higher age groups than among men or younger women.

Table A. Descriptive characteristics of the sample by age and gender

|  | *80 – 84* | | *85-89* | | *90+* | |
| --- | --- | --- | --- | --- | --- | --- |
|  | *Men* | *Women* | *Men* | *Women* | *Men* | *Women* |
|  | *M (SE) or %* | | | | | |
| **Living arrangement** |  |  |  |  |  |  |
| Coresidential partnership | 73.2 | 29.5 | 54.6 | 13.0 | 43.2 | 4.0 |
| Living-apart-together partnership | 5.2 | 5.8 | 10.9 | 3.0 | 7.0 | 1.9 |
| No partnership | 21.6 | 64.8 | 34.5 | 84.0 | 50.0 | 94.1 |
| **Social network** |  |  |  |  |  |  |
| Size (0-4) | 3.1 (0.1) | 3.2 (0.1) | 3.2 (0.1) | 3.2 (0.1) | 2.9 (0.1) | 2.8 (0.1) |
| Children and grandchildren | 73.6 | 79.5 | 77.2 | 77.6 | 67.6 | 72.2 |
| Siblings | 13.7 | 17.0 | 11.1 | 15.3 | 7.8 | 9.8 |
| Other family members | 27.0 | 30.3 | 29.0 | 42.7 | 36.9 | 40.3 |
| Friends | 14.4 | 20.6 | 9.7 | 13.4 | 8.1 | 11.0 |
| Acquaintances | 12.0 | 17.2 | 18.2 | 17.6 | 17.8 | 16.4 |
| **Education (ISCED 2011)** |  |  |  |  |  |  |
| Low | 8.7 | 34.0 | 10.5 | 42.9 | 10.1 | 42.7 |
| Intermediate | 56.4 | 54.7 | 52.5 | 48.1 | 59.5 | 48.2 |
| High | 34.8 | 11.3 | 37.0 | 9.0 | 30.4 | 9.1 |
| **Well-being** |  |  |  |  |  |  |
| Loneliness |  |  |  |  |  |  |
| Never or almost never | 84.9 | 74.4 | 73.6 | 69.5 | 64.6 | 64.3 |
| Sometimes | 11.7 | 20.4 | 19.5 | 23.2 | 26.2 | 25.0 |
| Often | 1.8 | 2.8 | 3.9 | 4.7 | 6.1 | 6.7 |
| Always or almost  always | 1.6 | 2.4 | 2.9 | 2.6 | 3.1 | 4.1 |
| Depression (0-4) | 0.8 (0.1) | 0.9 (0.1) | 0.8 (0.1) | 1.0 (0.1) | 1.0 (0.1) | 1.1 (0.1) |
| Total | 58.9 | 48.6 | 30.0 | 31.2 | 11.1 | 20.2 |
| N | 396 | 577 | 202 | 370 | 75 | 240 |

Note: NRW80+; n=1,860; weighted data.
