# Supplementary material for: Social relationships, living arrangements and loneliness
Source: Z Gerontol Geriatr. 2021 Aug 20;54(Suppl 2):120–5. doi: 10.1007/s00391-021-01960-1 (PMC8551096; doi:10.1007/s00391-021-01960-1)
Supplement: Supplementary file 2 — Supplement 2: Table B. Results of ordered logistic regression for loneliness [file 391_2021_1960_MOESM2_ESM.docx]

**Supplement 2**

*Complete results of Table 2 including control variables*

Table B. Results of ordered logistic regression for loneliness

|  | Model 1 | | Model 2 | |
| --- | --- | --- | --- | --- |
| Variable | ß (SE) | 95% C.I. | ß (SE) | 95% C.I. |
| **Living arrangement**  *(Ref. No partner)* |  |  |  |  |
| Coresidential partnership | -1.39*** (0.17) | -1.73 -1.05 | -1.25*** (0.17) | -1.58 -0.91 |
| LAT partnership | 0.14 (0.30) | -0.45 0.74 | 0.20 (0.30) | -0.40 0.80 |
|  |  |  |  |  |
| **Social network** |  |  |  |  |
| Size | - | - | -0.24** (0.07) | -0.37 -0.10 |
| Children and grandchildren | -0.24 (0.16) | -0.56 0.08 | 0.14 (0.20) | -0.26 0.53 |
| Siblings | -0.26 (0.25) | -0.74 0.23 | -0.08 (0.26) | -0.59 0.43 |
| Other family members | -0.13 (0.15) | -0.42 0.16 | 0.08 (0.15) | -0.21 0.38 |
| Friends | -0.19 (0.22) | -0.62 0.24 | -0.02 (0.22) | -0.46 0.42 |
| Acquaintances | -0.33 (0.20) | -0.73 0.07 | -0.14 (0.21) | -0.56 0.27 |
|  |  |  |  |  |
| **Controls** |  |  |  |  |
| Depression | 0.73*** (0.07) | 0.60 0.87 | 0.73*** (0.07) | 0.60 0.86 |
|  |  |  |  |  |
| Age | 0.03* (0.01) | 0.00 0.06 | 0.03* (0.01) | 0.00 0.06 |
|  |  |  |  |  |
| Gender *(Ref. Men)* | -0.11 (0.16) | -0.43 0.20 | -0.08 (0.16) | -0.39 0.23 |
|  |  |  |  |  |
| Education *(Ref. Low)* |  |  |  |  |
| Intermediate | 0.11 (0.19) | -0.27 0.48 | 0.12 (0.19) | -0.25 0.49 |
| High | 0.11 (0.24) | -0.36 0.58 | 0.15 (0.23) | -0.31 0.61 |
| Cut 1 | 3.83 (1.28) | 1.30 6.36 | 3.55 (1.28) | 1.02 6.07 |
| Cut 2 | 5.83 (1.29) | 3.28 8.38 | 5.56 (1.29) | 3.01 8.11 |
| Cut 3 | 6.85 (1.32) | 4.22 9.48 | 6.59 (1.33) | 3.95 9.22 |
| F | 19.32*** |  | 19.34*** |  |
| N | 1860 |  | 1860 |  |

Note: NRW80+; weighted data; * p<0.05; ** p<0.01; *** p<0.001.
